# Evolution and Expression Divergence of E2 Gene Family under Multiple Abiotic and Phytohormones Stresses in* Brassica rapa*

**DOI:** 10.1155/2018/5206758

**Published:** 2018-08-27

**Authors:** Nadeem Khan, Chun-mei Hu, Waleed Amjad Khan, Emal Naseri, Han Ke, Dong Huijie, Xilin Hou

**Affiliations:** ^1^State Key Laboratory of Crop Genetics and Germplasm Enhancement, Ministry of Science and Technology/College of Horticulture, Nanjing Agricultural University, Nanjing 210095, China; ^2^New Rural Research Institute in Lianyungang, Nanjing Agricultural University, 210095, China

## Abstract

To understand ubiquitination mechanism, E2s (ubiquitin conjugating enzymes) have crucial part as they play a major role in regulating many biological processes in plants. Meanwhile,* Brassica rapa* is an important leafy vegetable crop and therefore its characterization along with the expression pattern of E2s under various stresses is imperative. In this study, a total of 83 genes were identified in* B. rapa* and were classified into four different classes based on domain information. Here, we analyzed phylogenetic relationships, collinear correlation, gene duplication, interacting network, and expression patterns of* E2* genes in* B. rapa*. Furthermore, RT-PCR analysis for 8 multiple abiotic and hormone treatments (namely, ABA, GA, JA, BR, PEG, NaCl, and heat and cold stress) illustrated striking expression pattern under one or more treatments, speculating that these might be stress-responsive genes. The* cis*-elements and interaction network analyses implicate valuable clues of important function of* E2* genes in development and multiple stress responses in* B. rap*a. This study will further facilitate functional analysis of E2s for improving stress resistance mechanism in* B*.* rapa*.

## 1. Introduction

Among the eukaryotes, ubiquitination is considered one of the important types of posttranslational modification of proteins. It plays crucial role in plant developmental aspects with holding imperative association, such as regulating a number of biological process [1], responding to plant biotic and abiotic factors, light regulation, phytohormones, flowers developments, and others several key factors [2–5]. The complex nature of ubiquitination generally is categorized into three types of enzymes, namely, ubiquitin-activating enzyme (E1), ubiquitin conjugating (UBC) enzyme (E2), and ubiquitin-ligase enzyme (E3) as with target protein the ubiquitin binds covalently during this process [6]. Except for ubiquitin E1, the other two E1 and E2 enzymes are linked in an ATP-dependent manner and are activated ubiquitin, transfer targeted protein aided by E3 after passing it to active-site cysteine [6]. The targeted protein can be also modified through sequential ubiquitination cycles as the additional ubiquitin is further ligated into initial ubiquitin, which ultimately forms a polyubiquitin chain, helping them in targeted protein modification [6]. For generating other biological activities, then the substrates can be degraded. Due to the attachment of ubiquitin with targeted protein, E2 is considerably important and plays a crucial role in ubiquitination [6].

On a global scale the crop productivity is always uncertain due to climatic changes and series of other biotic-abiotic factors that damage the plant growth and production substantially and lead to severe crop losses. Some of the recent studies highlighted that involvement of* E2* genes family exited as multifunctional and playing key parts in various physiological activities. In several crops, such as in grapes 43 [7], tomato 59 [8], rice 39 [9], maize 75 [10], Carica papaya 34 [11], and Arabidopsis 41 [12], genes were identified, respectively, as during the process of evolution the number of genes of E2 increases with the complex nature and the development of organism [13]. For example, fewer genes ≤ 20 in algae were found in ancestral eukaryotes compared to certain plants and animals > 40 [14]. The increase in numbers and the diversification of E2s are typically governed and have been associated with the eukaryotic evolution [15]. The biological diversity drives mainly with the subsequent identification of novel molecular functions [16]. However, functional analysis of E2s family in higher plants is limited on the basis of extensive studies. For example, in transgenic Arabidopsis, peanut, and soybean the* E2* genes respond to drought stress and salt tolerance [17–19], while in mung bean VrUBC1 were found to respond to the osmotic stress [20]. Tolerance of UV stress and as well the activation of floral repressor genes such as AtUBC1 and AtUBC2 is important [21]. The* E2* genes family are specifically important from the above studies and their further characterization will shed light on their action mechanism. Furthermore, this may help in the distribution of ubiquitin-proteasome with fundamental understanding.

Chinese cabbage belonging to genus* Brassica* is an important leafy vegetable crop being grown worldwide. Genotype Chiifu 401-42 (Chinese cabbage) was recently sequenced and assembled. Since the divergence of* B. rapa* from Arabidopsis 13 to 17 MYA it experienced a whole genome triplication (WGT) event and the data exhibits a close evolutionary relationships [22, 23]. During this study, we carried out genome-wide analysis of BraE2 and its expression divergence patterns. The conserved motifs, coregulatory network, gene duplications, and in silico transcriptome for various tissues were conducted. On the other hand, we also investigated and validated the BraE2 expression patterns under multiple abiotic and hormone treatments. We attempted a complete picture of BraE2 in* B. rapa* and these results will further provide guidelines for functional analysis of BraE2 each genes.

## 2. Material and Methods

### 2.1. E2 Ubiquitous Conjugating Enzymes Identification

The* B. rapa* sequences were downloaded from the database (http://brassicadb.org/brad/), while for other species rice [9] (http://rice.plantbiology.msu.edu/), tomato [8] (https://solgenomics.net/organism/Solanum_lycopersicum/genome), Maize [10] (https://www.maizegdb.org/),* Vitis vinifera* [7], and* Carica papaya *[11] sequences were downloaded based on the reported studies. From Pfam database (https://pfam.sanger.ac.uk/) the E2 UBC domains (PF00179) were first downloaded and* Hidden Markov Model* (HMM) search was carried out in* Brassica* genome with default parameters. After identifications, we verified these potential sequences using NCBI database (https://www.ncbi.nlm.nih.gov/) and SMART (http://smart.embl-heidelberg.de/). The theoretical index (pI), GRAVY (Grand Average of hydropathicity), protein length (aa, and molecular weight were examined through ExPASy protparam (http://web.expasy.org/protparam/) and, for subcellular predication, we explored WoLF PSORT server (https://wolfpsort.hgc.jp/).

### 2.2. Phylogenetic Analysis and Gene Duplications

To study the evolutionary relationship of BraE2, we performed multiple sequence alignment using CLUSTALW with default parameters in MEGA 7.0. A phylogenetic tree was constructed using Maximum Likelihood Method (MLM) with Jones-Taylor and Thornton amino acid substitution method (JTT) using MEGA 7.0 [24] and was performed with 1000 bootstrap replication. We also examined the* Ks/Ka* (synonymous and nonsynonymous) values among the duplicated genes with the help of MEGA 7.0. The coding sequences of duplicated pairs of BraE2 were first aligned by following the Nei and Gojobori model performed in MEGA 7.0 [24].

### 2.3. Motif and Gene Structure Analysis

The conserved motif was analyzed through MEME (http://meme-suite.org/) a limit of 20 motifs with minimum width ranges from 10 and maximum 120, and the others parameter were set as default. For genes structure analysis, we explored Gene Structure Display Server (http://gsds.cbi.pku.edu.cn/) (GSDS) and the exon-intron organization of BraE2 was determined by comparing the coding and its corresponding genomic sequences.

### 2.4. Chromosomal Localization and Gene Syntenic and Promoter Analysis

All the information for each gene of BraE2 was collected from the* B. rapa* database (http://brassicadb.org/brad/) and the images were drawn through Mapchart [25]. Syntenic genes were also identified among the three subgenomes (LF, MF1, and MF2) of* B. rapa *database. All the identified BraE2 ubiquitin enzymes, 15 kb promoter sequence, were analyzed through PlantCARE database (http://bioinformatics.psb.ugent.be/webtools/plantcare/html/) for the identification of* cis*-regulatory elements [26].

### 2.5. Expression Pattern Analysis and Interacting Network of BraE2 Genes

For expression profiling of BraE2 among various five tissues (root, stem, leaf, flower, and silique), we analyzed* B. rapa *accession (Chiifu-401-42). Based on the previously reported RNA-seq data [27] for gene expression patterns were utilized and the fragments per kilobase of exon model per million mapped (FPKM) values were transformed into log2. Finally, heat maps were generated for all the BraE2 and for their paralogous pairs using omicshare tools (http://www.omicshare.com/). For interaction network, we explored string (https://string-db.org/).

### 2.6. RNA Isolation and qRT-PCR Analysis

We isolated the RNA from the treated leaves of* B. rapa* with the help of RNA kit (RNA simply total RNA kit; Tiangen, Beijing, China) according to the manufacturer's instructions. After that, for every RNA sample, we checked the quality and quantity by using an agrose gel. With the help of Prime Script RT reagent kit (TaKaRa) the RNA was then reversely transcribed into cDNA. For qRT-PCR analysis, gene specific primers were designed by Beacon Designer 8.0 and are listed in SupplementaryTable 1). The reactions were performed using Step one plus Real-Time PCR System (Applied Biosystems, Carlsbad, CA), with the help of the following parameters: 94°C for 30 s, 40 cycles at 94°C for 05 s, and 60°C for 15 s and 72°C for 10 s. In order to check the specificity of the amplification melting curve was generated with following parameters: 61 cycles at 65°C for 10 s. The relative gene expression values were calculated using the comparative Ct value method [28].

### 2.7. Plant Materials and Multiple 8 Treatments

For this experiment, we used a typical Chinese cabbage cultivar Chiifu 401-42. It has been mostly used for research studies, mainly due to its complete whole genome sequencing. In greenhouse of Nanjing Agricultural University, we cultivated Chinese cabbage in potting soil with following controlled environmental conditions as follows: 65-70% humidity, light 16 h/25°C, and dark 8 h/20°C. After a one-month interval, when the seedlings reached five-leaf stage they were subjected to multiple abiotic and hormone stresses under continuous time intervals (1, 6, and 12 hrs). Meanwhile, for multiple hormone treatments plant was treated with four different types such as ABA (100 *μ*M), GA (100 *μ*M), JA (50 *μ*M), and BR (50 *μ*M). Salt stress plants were subjected to 250 mM, while drought stress pots were irrigated with 15% (w/v) polyethylene glycol under normal growth conditions. For salt and osmotic treatments, irrigation solution was kept standing for 30 mins, respectively. Cold and heat stress plants were exposed to 4 or 38°C, while other growth parameters were set as discussed above. All treatments were based on three biological replicates at the end leaf samples which were frozen immediately in liquid nitrogen and stored at −70°C for further analysis.

### 2.8. Pearson Corelation

The PCCs values for transcriptomic data and qRT-PCR were performed in excel sheet 2013 based on the paralogous pairs [29].

## 3. Results

### 3.1. Identification and Bioinformatics Analysis of UBC Genes in* B. rapa*

**Figure 1:**
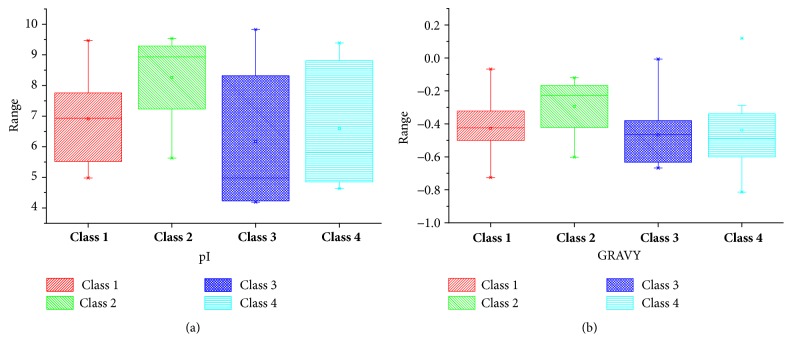
**(a)** Indicating the* pI* values among different subclasses of BraE2.** (b) **Showing the grand average of hydropathicity (GRAVY) among different classes of BraE2.

In the present study, we explored multiple bioinformatics resources for the identification of UBC (Ubiquitin Conjugating) genes in* B. rapa. *A total of 83 genes were identified through genome-wide analysis by using HMM and BLAST search methods. All these candidate genes were designated as BraE2-1-BraE2-83 according to generic order and were classified into four major classes based on domain information. The characteristic analysis of these 83 genes including Gene ID, cDNA (bp), Chr: Start-End point, Exon, Subcellular predication, pI, GRAVY (Grand Average of hydropathicity), Protein length (aa), and MW (kDa) were analyzed and are listed in (Supplementary, Table. 2) for each protein. The number of amino acid were markedly varied from 111 to 1662 in length, with the corresponding molecular weight (MWs) ranging from 12.38 to 185.26 in kDa, respectively, and the theoretical isoelectric points varied from 4.19 (BraE2-75) to 9.83 (BraE2-40) which were further shown in Figure 1(a) for different classes. According to the stability index measure most of the proteins were unstable with hydrophilic in nature; however two proteins (BraE2-13-BraE2-4) from Class 1 were hydrophobic, suggesting their stability (Figure 1(b)). Majority of genes were located in different specific-organelles such as mitochondria, nucleus, cytosol, endoplasmic reticulum, plasma membrane, and other secretory pathways, whereas they were regulated in variable microenvironment.

### 3.2. Phylogenetic Analysis and Structure of BraE2 Genes

**Figure 2:**
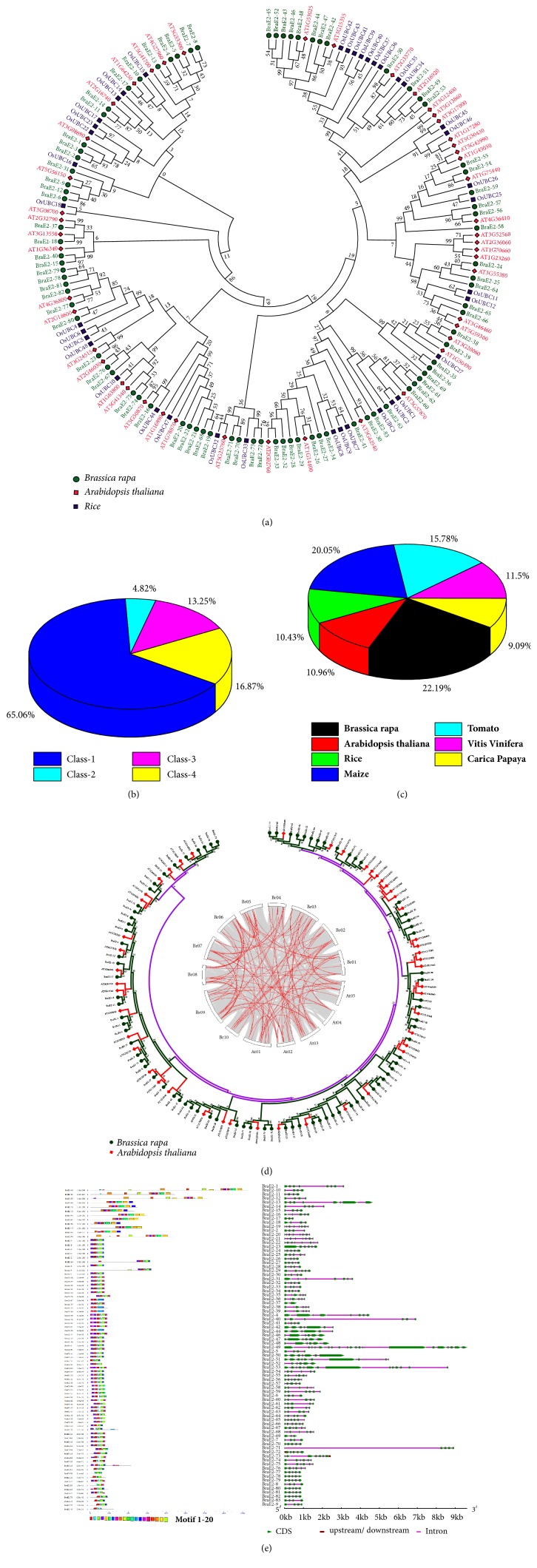
**(a)** Phylogenetic relationships of BraE2 among three species* B.rapa, A.thaliana*, and rice. The phylogenetic tree was constructed by MEGA 7 using the Maximum Likelihood Method (1000 bootstrap). Genes of different species are marked with different colors.** (b) **Relative classification patterns of BraE2 family based on the number of genes among different classes.** (c) **Showing classification pattern of* E2s* genes in different species.** (d)** Phylogenetic tree and Circos plotter family. (A) The phylogenetic tree was constructed by MEGA 7 using the Maximum Likelihood Method (1000 bootstrap). (B) The Circos plotter family between* B. rapa* and* A. thaliana* were elucidated by MCScanX program.** (e) **The conserved motif was constructed by MEME program. The intron, upstream/downstream, and CDS region are represented by pink, brown and line and green boxes, respectively. The bottom of the figure the relative position is proportionally displayed based on the kilobase scale.

To investigate the evolutionary relationship and functional divergence between BraE2 proteins and known BraE2s from other species, a maximum likelihood phylogenetic tree was constructed on the basis of the full amino acid of BraE2 family protein from* B. rapa*,* A. thaliana*, and rice (Figure 2(a)). The* BraE2* genes represent similarities on the basis of phylogenetic analysis and contain UBCc (Ubiquitin Conjugating Enzyme, E2 catalytic) superfamily domain. Furthermore, on the basis of domain information BraE2 family were further distributed into four major classes such as Class 1 contain only UBC catalytic domain, Class II (N-terminal extension), Class III (C-terminal extension), and Class IV contains (both N- and C-terminal extensions) [30, 31]. We found that Class I represents the highest 54 members, followed by Class IV, Class III, and Class II with 14, 11, and 4 members, respectively, and among all the classes highest 65. 06% proportion belonged to Class I (Figure 2(b)). We also calculated the proportion of genes in various species based on previous reported studies (Figure 2(c)). Notably, the* B. rapa *were found to have highest (22.19%) number of genes compared to other species such as maize (20.05%), tomato (15.78%), Vitis Vinifera (11.5%), Arabidopsis (10.96%), rice (10.43%), and Carica papaya (9.09%). On the other hand, we also a constructed a phylogenetic tree between* B. rapa* and* A. thaliana *with family circle plotter using MCScanX program (Figure 2(d)).

To better understand the structural diversity of BraE2 in* B. rapa, *we compared the intron/exon structure and conserved motifs (Figure 2(e)). The gene structures were obtained by comparing the genomics and ORF sequences. Moreover, each structure possessed a minimum of 1 and 21 maximum intron. Intriguingly, for Class IV the BraE2-49 genes were found to have highest 21 numbers of intron compared to other classes. For majority of genes the common pattern with 32.5% shared 3-4 numbers of intron/exon. In addition, for different classes of BraE2 the intron/exon was mostly similar in pattern for same classes and for those which were closely related members. There was only one gene, BraE2-37 with one intron. Furthermore, twenty conserved motifs were captured using MEME software. Surprisingly, most numbers of motifs 13 were found for Class IV (BraE2-49) gene. Motifs 1, 2, 3, and 4 were present in almost all the members of BraE2, while other motifs were detected in less than half of BraE2. The regulation patterns for most of the complex structure were tight among few members of the BraE2.

### 3.3. Collinearity Correlation of BraE2 among* A. thaliana* and* B*.* rapa *and Copy Number of Variation

**Figure 3:**
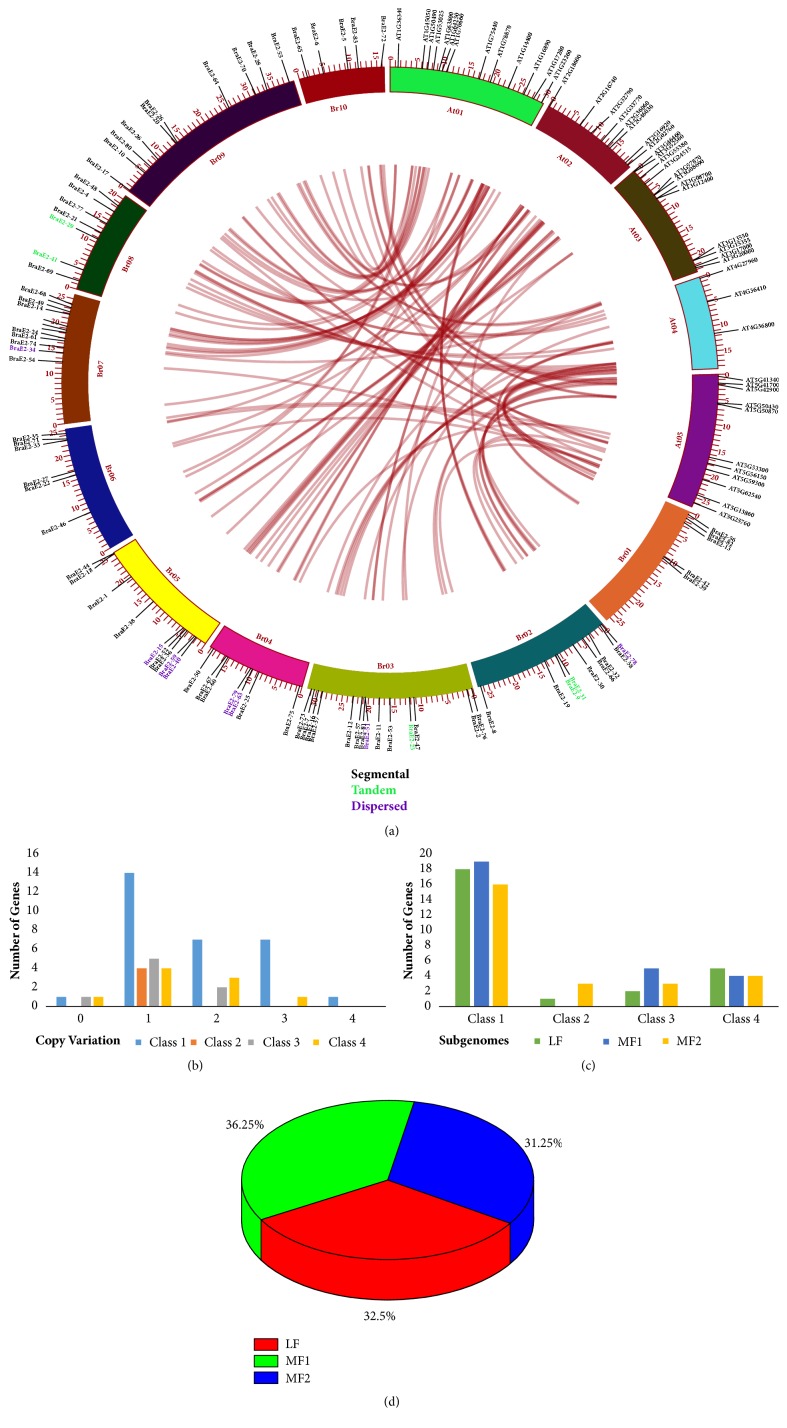
**(a) **The collinear correlation for all the genes of BraE2 was displayed between* B. rapa *and* A. thaliana.* The ten Chinese cabbage chromosomes (Br01-Br10) and the five* A. thaliana *chromosomes (At1-At5) are shown in different random colors. The illustration was drawn using Circos Software. For duplication types, tandem array was displayed with green color and dispersed with purple.** (b)** Showing copy number of variation among different subclasses of BraE2s.** (c)** Showing number of genes based on 4 different subclasses of BraE2s.** (d) **Showing the ratio of* BraE2* genes among three subgenomes of* B. rapa*.

We explored two things, collinear correlation between* B. rap*a and* A. thalia*na genes which were presented in (Figure 3(a)) and the copy number of variation during* B. rapa* specific WGT event. Interestingly, the highest number 14 for one-copy variation was found in Class I, followed by two- and 3-copy variation with 7 each, respectively, as shown in Figure 3(b) and Supplementary Table 3. The* B. rapa* genome basically contained three genomes, namely, LF (least fractioned genome), MF1 (medium fractioned), and MF2 (more fractioned). Based on these three genomes, we calculated the number of genes for different classes, although the highest number of genes 19 was found in MF1, followed by 18 in LFand 16 in MF2for Class I compared to other classes (Figure 3(c)). Overall, results showed that different three subgenomes were more similar with slight variations (Figure 3(d)) and ratio for MF1 was highest 36.25%, followed by LF 32.5% and MF2 31.25%.

### 3.4. Chromosomal Localization, Gene Duplication and Selective Pressure Analysis of BraE2

**Figure 4:**
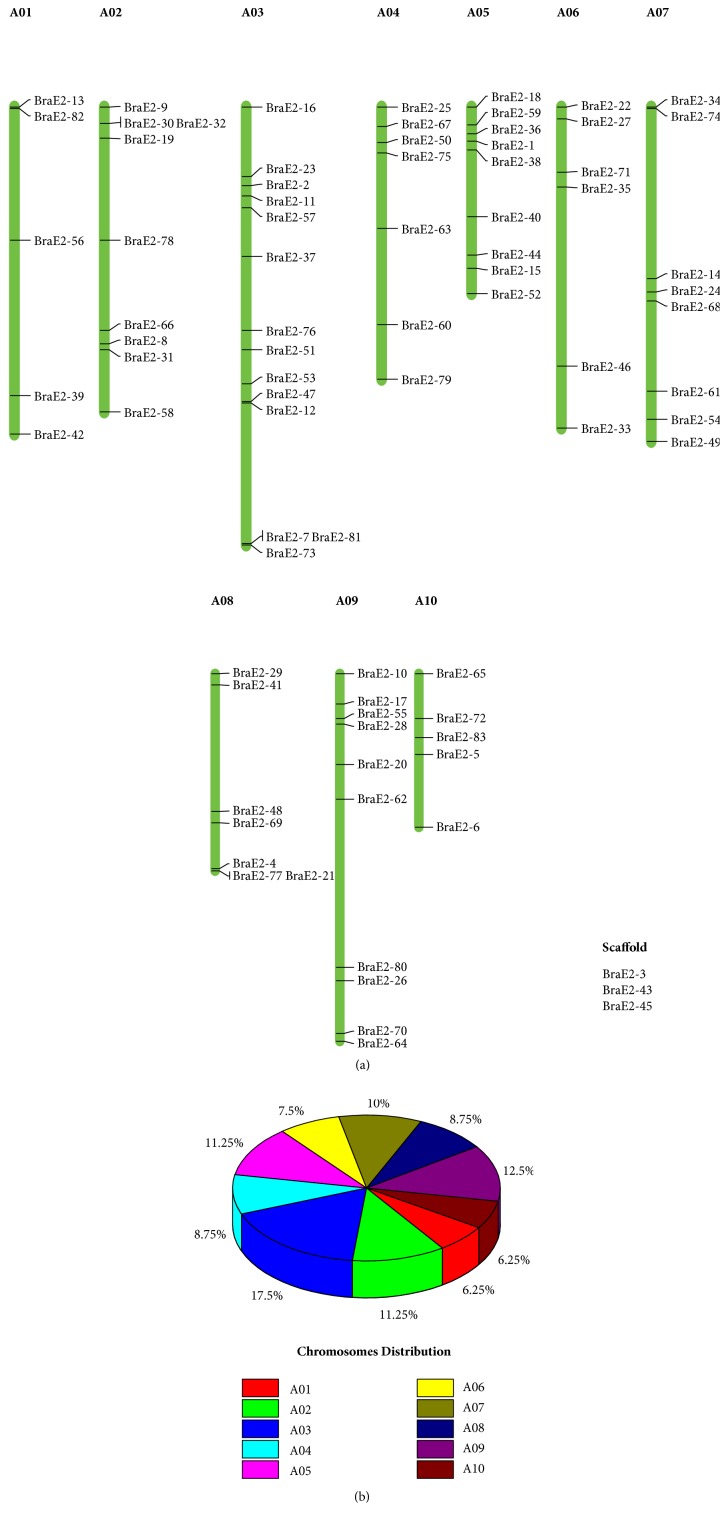
**(a) **Chromosome location of the BraE2 was obtained from the GFF file and displayed by using Mapchart. (b) The relative shares of different subclasses of BraE2 from A01 to A10 chromosomes of* B. rapa.*

The BraE2 chromosomes were distributed on all the ten (A01-A10)* B*.* rapa *genomes except from three genes (BraE2-3, BraE2-43 and BraE2-45) which was on scaffold (Figure 4(a)). The distribution pattern of BraE2 were highly varied, majority of genes 13 were found on A03, followed by 10 on A09, whereas chromosomes A02 and A05 contains 9 number of genes each respectively. The overall ratio of these chromosomes (A01-A10) for BraE2 are further presented in (Figure 4(b)).

To explore the functional diversification of protein and duplication analysis are significantly important in gene family [32]. In this study, we analyzed mainly two types of duplication (Segmental and tandem) to examine the contribution of duplication events in this family (Supplementary, Table.  4). Based on the paralogous pairs of* B.rapa* genomes, we calculated* Ka/Ks* ratio for ten pairs of segmental and one of tandem array. The results showed that all the pairs had a less than 1.00 values, which indicated that these pairs were purifying in nature. The rate of divergence for all the duplicated pairs with an average of 7.37 (MYA), suggesting that their divergence occurred during* Brassica* triplication (5~9 MYA) event. Furthermore, with the help of MCScanX program, we identified the types of duplication.

### 3.5. Gene Expression Pattern in Various Tissue and Syntenic Paralog Pairs Patterns in* B. rapa*

**Figure 5:**
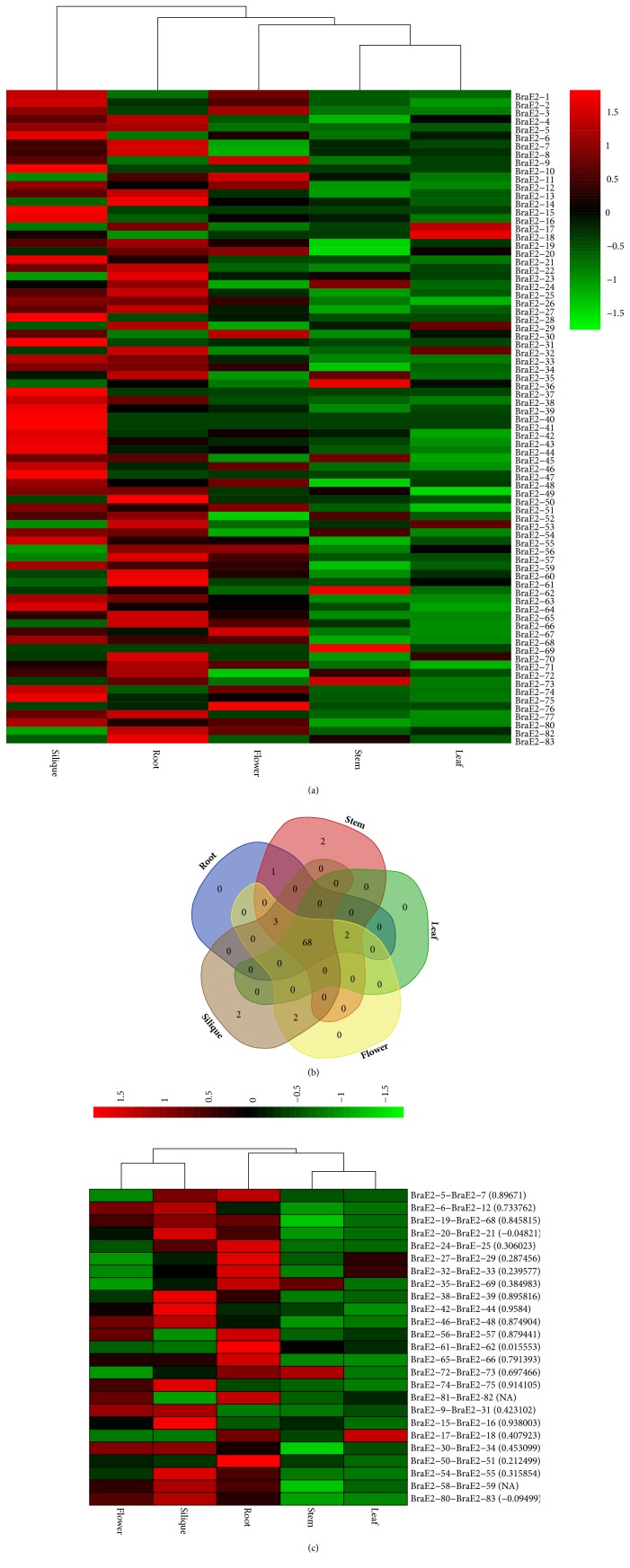
(a) Heat map of expression profiles (in log2-based FPKM) for BraE2s in the five tissues of stem, root, leaf, flower, and silique. The expression levels are exhibited by the color bar. (b) Venn diagram analysis of the tissue expression of BraE2s.** (c).** Heat map of expression profiles for BraE2 25 paralogous pairs in the five tissues of stem, root, leaf, flower, and silique. The Pearson correlation coefficient (PCC) is also displayed in bracket while NA indicate no available results for PCC.

Since no ubiquitous conjugating enzymes have been documented in* B. rapa *previously, we analyzed the expression patterns of BraE2 under 5 various organs (roots, stems, leaves, flowers, and siliques) of* B. rapa *(Figure 5(a). Supplementary Tables 5 and 6). A heat map for expression patterns was generated by displaying the expression profiles in clustered for BraE2. Majority of the genes showed a striking expression pattern for different classes, whereas a few of them exhibited a similar expression pattern. Moreover, some of the genes, about 11.08%, showed no expression pattern in any tissue, and the rest of them showed a significant variation in expression pattern in one or more tissues. The tissue clustered expression pattern was also exhibited (Figure 5(b)), and two genes from stem and siliques each, respectively, were expressed, suggesting that these genes may play a specific role in the relevant tissues.

We also investigated trends of expression pattern for 25 paralogous pairs (Figure 5(c)). These paralogous pairs showed a high alteration in expression level in five tissues. Most of these genes showed a high expression patterns, speculating that these paralogous pairs might have similar function. Additionally, the PCC values for paralogous pairs across five tissues were also calculated and a total of 11 pairs showed a (> 0.6) PCC, suggesting a positive correlation. In general, we can infer a positive close correlation between two factors with (> 0.6) PCC. Such positive correlation among BraE2 pairs probably implicates functional conservation or subfunctionalization after duplication. There were two pairs such as BraE2-20-BraE2-21 and BraE2-80-BraE2-83 with negative PCC and two pairs with no PCC (BraE2-81 BraE2-82, BraE2-58 BraE2-59), implicating that these pairs due to pseudogenization might loss function.

### 3.6. Coregulatory Networks and Expression Profiling of BraE2 Genes in Response to Multiple Abiotic/Phytohormones Treatments

**Figure 6:**
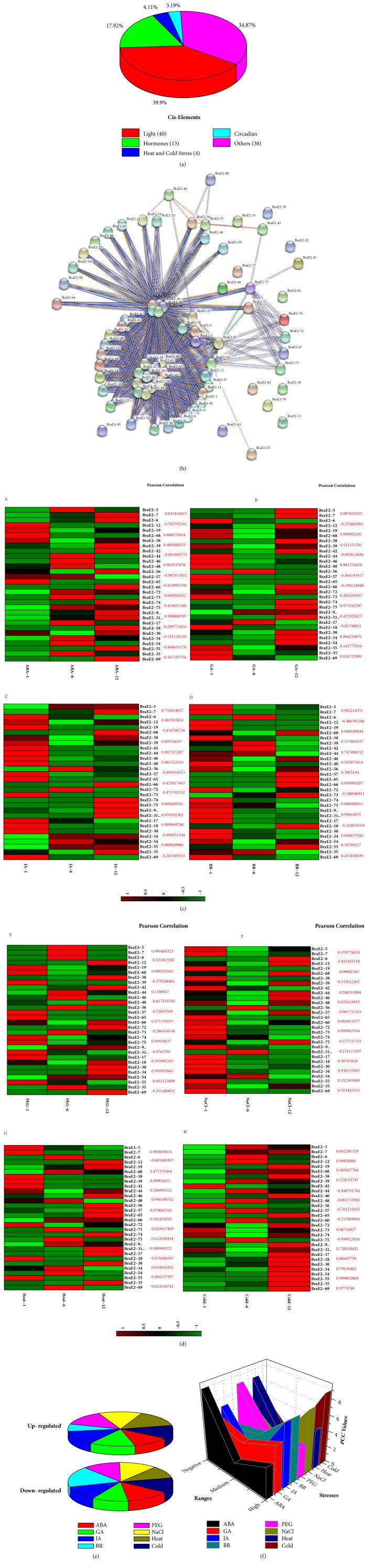
**(a) **Showing the ratio of different* cis*-element.** (b)** The coregulatory network for BraE2s was presented by STRING sever.** (c and d)** Expression analysis of the* BraE2* genes under multiple abiotic and hormone treatments in* Brassica rapa *(A-H). Heat map representation of the* BraE2 *genes for abiotic/hormone treatments (namely, ABA, GA, JA, BR, PEG, NaCl, heat, and cold stress). Each pair of BraE2 along their PCC values is also displayed.** (e) **Showing the up- and downregulated genes in response to multiple abiotic/hormone treatments.** (f)** Showing the PCC values under response to multiple abiotic/phytohormones treatments.

To analyze the* cis*-regulatory elements, we utilized online tool PlantCARE for the identification of* BraE2 *genes in* B. rapa. *Based on our results, we make five categories such as Light, Hormones, Heat and Cold, and Circadian while each category contains related responsive elements (Figure 6(a)). In addition, we calculated the number of genes and find their percentage among five categories. A high number of genes (39.9%) were found in light category, common light responsive elements with 40 common* cis*-regulatory elements were identified (ACE, GAP, LAMP, GTI, GATA, G-Box, ATI, and others), further summarized in Supplementary Table 7. Most of the common regulatory elements were dominated by another category and about 34.89% genes were involved in both biotic and abiotic stresses and common* cis*-regulatory types were ARE, Wun-Motif, MBS, TC-Rich repeats, and others. For hormones category, 17.92% genes participated and 13 types such as ABRE, CGTCA, TCA, and GARE motif were found in the promoter regions of BraE2, suggesting that it could affect the expression levels of* BraE2 *genes in* B*.* rapa. * A substantial number of light responsive, hormones, heat and cold stress, and other important elements were observed in the promoter sequences of BraE2. This clearly indicated their probable role in both biotic-abiotic factors and hormonal pathways.

The biological and signaling transduction pathway are typically governed by genes through interaction network. To understand gene family function, the investigation of potential network is considerably important [33]. To further elucidate the interaction network of BraE2 family, proteins-protein interaction were constructed with the help of STRING software. Most of the genes showed a close and condense relationship with each other except from a few genes as shown in (Figure 6(b)). Among all the* BraE2* genes showed a very dense correlation, suggesting that these genes are involved in several fundamental mechanisms and are further regulated by many down/upstream genes.

To study genes function its expression profile provides a useful information with valuable clues for understanding it [34]. Recent studies have suggested that* BraE2* genes have been implicated in plant responses to signaling and abiotic stresses [9, 19, 35], and the presence of stress-responsive* cis*-elements in the promoter of the* BraE2* genes speculated that their involvement in* B. rapa* is a response to different stresses. To confirm this speculation, the transcriptional expressions of the 15 pairs of BraE2 were analyzed in* B. rapa* by qPCR after application of multiple abiotic and hormone stresses such as, ABA, GA, JA, BR, PEG, NaCl, and heat and cold stress. Heat map was generated in response to eight multiple treatments for transcript expression fold change as shown in Figures 6(c) and 6(d) and Supplementary Table 8. Most of the putative genes were highly expressed and showed high striking expression patterns. Majority of the genes (55.55%) were upregulated and (44.44%) downregulated in response to heat treatment, followed by GA (52.22%) upregulation and (47.77%) downregulation. Interestingly, both PEG and NaCl showed similar expression patterns as 50% were up- and downregulated. Noticeably, BR were found to be sensitive, as most of the genes (74.44%) were downregulated, in case ABA and cold treatment minor changes were exhibited in the expression patterns as 42.22% and 46.67% were upregulated (Figure 6(e)). On the other hand, based on the results of relative expression values, we analyzed the correlation and regulatory network among the selected 15 pairs of BraE2. For correlation, we categorized the PCC values into three sections such as High (>0.6), Medium (>0 and < 0.5), and Negative (< 0). For cold stress, 9 PCC values were higher among all, followed by JA, BR, and NaCl with 8 PCCs each value observed, respectively. These results indicate and signify their close relationship among each other, whereas for ABA treatment most of the 9 PCC values were negative which suggested its contrasting nature to other treatments (Supplementary Table 9 and Figure 6(f)).

## 4. Discussion

### 4.1. Identification, Phylogeny, and Gene Duplication of BraE2 Family

In many aspects of plant growth and development, ubiquitin conjugating enzymes are considerably important and crucial to variety of plant stress responses. In the present study, a total of 83* BraE2* genes were identified with UBC domain in* B. rapa* genomes. Based on the comparison with other species, this number was higher as shown by previous reported studies such as 75 identified in maize [10], 59 in tomato [8], 50 in human [36], 41 in Arabidopsis [12], and 39 in rice [9]. The variation in the number of* BraE2* genes shows speculation that during the course of evolution BraE2 family had underwent functional divergence. The BraE2 were further subdivided into four different classes, namely, Class I-IV based on the domain information with respect to N- and/or C-terminal extensions [30, 31]. Majority of* BraE2* genes were retained, particularly in Class I as there were 14 one-copy variants and 7 each for two or three copy variations compared to others classes of BraE2 in* B. rapa*. As a result a high number of BraE2 members were retained in* B. rapa *genome, after whole genome duplication event (WGD). Expansion of gene family and the adaptation of gene function are reliable on gene duplication as per environmental conditions. Variations in either structural features of coding sequences or amino acid leads to the functional diversification [37]. To understand duplication events, here we analyzed the expansion mechanism of BraE2 family. The results showed that 10 pairs from segmental duplication and one pair of tandem duplication were identified based on paralogous pairs, suggesting that segmental duplication contributed to the expansion of BraE2 family. Our results are further in agreement with the gene dosage hypothesis [38]. In addition, we also calculated* Ka/Ks* ratio of these 11 pairs. All the pairs of* BraE2* genes indicated less than 1.00 value, strongly implicating its purifying selection in nature. Furthermore, the BraE2 diverged 7.37 MYA during the specific Brassica WGT event. These results suggested that, to overcome the selective pressure for their survival needs the BraE2 duplicate early, which signify their diverse functions in nature. In the present study, a phylogenetic tree was constructed among three species such as* B. rapa*,* A. thaliana*, and rice. Our resulted classification for BraE2 family was consistent with domain information. To gain insight into the structural diversity of the BraE2 family, gene structure and conserved motifs were also analyzed. It is well understood that the diversity among species mainly happens through genes organization in the genome [37]. The structure and function of molecules in a system can be understand through patterns of motif in the nucleotide or protein sequences [39]. The patterns for most of the genes were similar in nature and were dominated by 3-4 numbers of intron. The regulation patterns for conserved motif were tight and specifically motif 1, 2, 3, and 4 were common in the BraE2 family. In addition, the process of tissue expression divergence is mainly associated with expansion of family and the duplication types in the neofunctionalization or subfunctionalization models [40–42].

### 4.2. Expression Divergence, Multiple Abiotic/Hormone Treatments, and Interaction Network

In this study, we also examined the tissues-specific expression patterns of BraE2, and majority of genes showed a high striking expression patterns in all the five tissues or at least several. Some of the genes were expressed in similar patterns, suggesting their common importance in function of plants. In addition, there were few genes that showed tissue-specific patterns, indicating that they might have acquired new functions for plant improvement. However, there was divergence in the expression pattern among the duplicated paralogous pairs which further suggested that after duplication in the evolutionary process some of them may acquire new functions. To understand the expression mechanisms of BraE2s, we identified common* cis*-regulatory elements in the promoter regions of BraE2s. The results, provided valuable information as majority of genes, were involved in light regulation, hormones, and other key biotic-abiotic factors. Based on the results of RNA-seq data and cis-elements, we performed qRT-PCR after multiple abiotic and hormone treatments. We selected 11 pairs of duplicated types and 4 pairs randomly among* BraE2* genes to explore the expression profile of BraE2. The ratios for majority of treatments were upregulated, such as for heat stress (55.55%), GA (52.22%), PEG, and NaCl stress (50%); however BR was more sensitive as a large number (74.44%) of genes were downregulated. Furthermore, based on PCC values, cold stress was among the highest with (9) PCC values (>0.6) which signify a close relationship and their expression profiles resulted in (46.67%) upregulation in genes. We further speculated that the function of genes was enhanced and expanded through genes duplication. Though, functional analysis will confirm and determine the pivotal role of BraE2. The expression levels of AhUBC2 in peanut plants were regulated by PEG, NaCl, ABA, and physiological stress [18]. In response to biotic-abiotic stresses and cellular responses from wild rice, OgUBC1 were reported [35]. In Arabidopsis, through application of salt stress AtUBC32 were greatly influenced and were reported to play a role in the BR (brassinosteroid) salt stress tolerant plant [19]. In plants, response to abiotic stresses and hormone-signaling are integral and it has been reported that in hormone-mediated stress responses the E2s play a major role [3–5]. These result speculated that E2s are critical for multiple stress responses in various species. Therefore, taken together, our results speculated that* BraE2* genes family might be contributing into functional divergence and playing a critical role during abiotic/hormone stresses.

## 5. Conclusion

In this study, a total of 83 members of BraE2 were identified and were classified into four major classes based on the domain information and phylogenetic tree. To predict the functional characteristics of BraE2, we analyzed the physicochemical properties along with gene duplication analysis. RNA-seq data and qRT-PCR results presented significant roles of BraE2 in the growth, development, and resistance mechanism of* B. rapa* under various stresses. The intron-exon distribution for most of the genes shared a pattern of junction, and gene duplications analysis showed that segmental duplication being major factor for the expansion and close evolutionary relationships of the gene family. Based on our results, as evidenced by gene expression analysis especially under various abiotic and hormone stresses, we hypothesize that* BraE2* genes show divergence in their function. Moreover, these results provide novel insight by providing a solid foundation for future functional dissection of BraE2 family in* B. rapa*.

## Data Availability

The data used to support the findings of this study are available from the corresponding author upon request.

## Supplementary Materials

Supplementary 1 
** Figure. Is. **Showing the PCC values for multiple treatments with values > 0.5.

Supplementary 2 
** Table. 1.** Sequences of the* BraE2* gene primers used for quantitative real-time PCR.

Supplementary 3 
** Table. 2.** The basic description of* BraE2 *genes in* Brassica rapa*.

Supplementary 4 
** Table. 3. **Identification of* BraE2 *syntenic genes between* A. thaliana *along with three subgenomes of* Brassica rapa*.

Supplementary 5 
** Table. 4.** The gene with outlier* Ka/Ks* values and estimated divergence time for syntenic pairs of BraE2.

Supplementary 6 
** Table. 5.** The FPKM values of* BraE2 *genes.

Supplementary 7 
** Table. 6.** Syntenic paralog pair of BraE2 with PC and FPKM values.

Supplementary 8 
** Table. 7.** *Cis*-elements of* BraE2* genes in* Brassica rapa*.

Supplementary 9 
** Table. 8.** Relative expression pattern of* BraE2* genes along with PC values with respect to various stresses by qRT-PCR.

Supplementary 10 
** Table. 9.** Pearson correlation coefficient of the stress-induced* BraE2 *genes whose PC is greater than 0.5.
