# Supplementary figures and images for: Evolution and Expression Divergence of E2 Gene Family under Multiple Abiotic and Phytohormones Stresses in Brassica rapa
Source: Biomed Res Int. 2018 Aug 27;2018:5206758. doi: 10.1155/2018/5206758 (PMC6129857; doi:10.1155/2018/5206758)

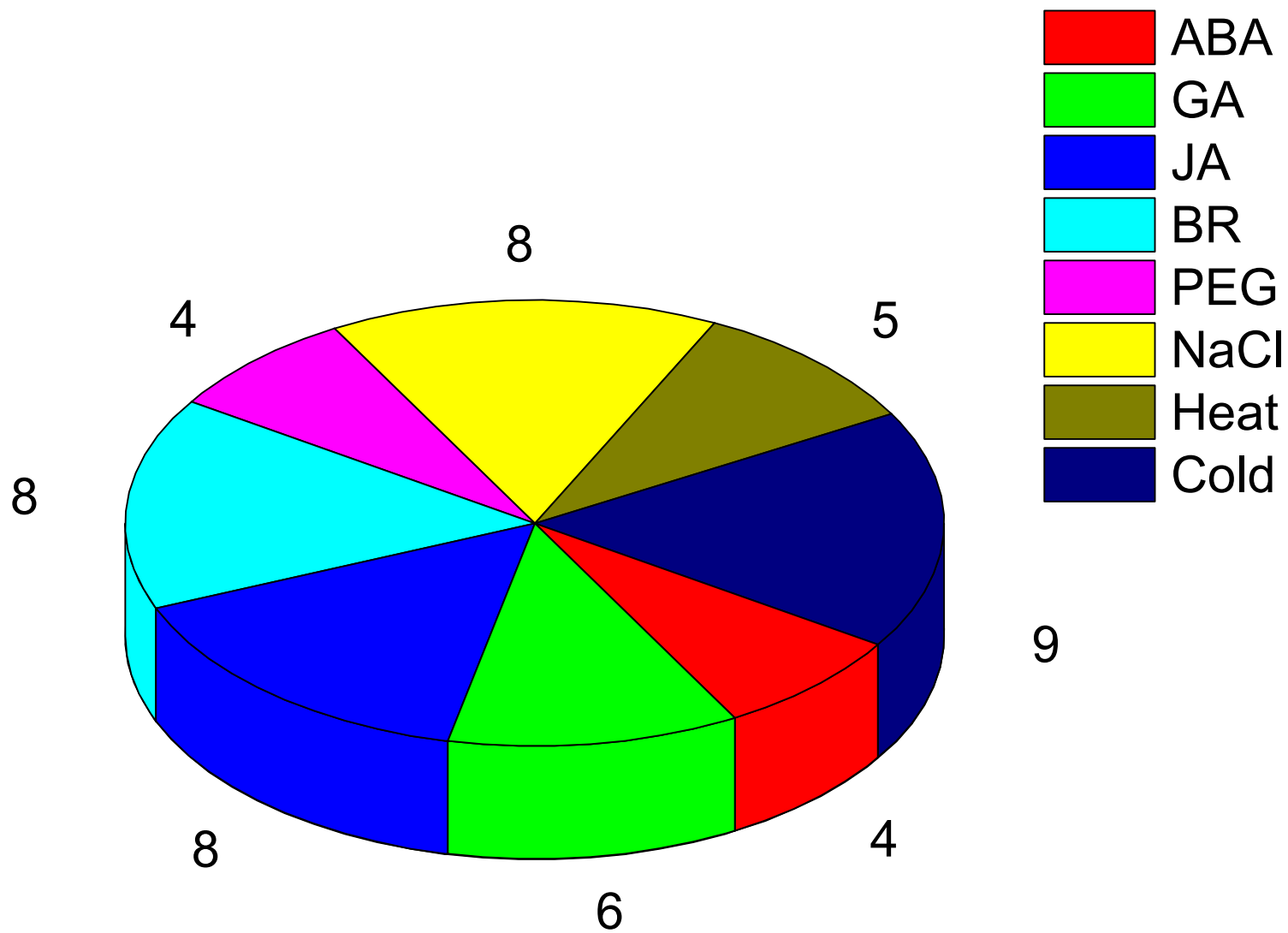

**PCC Greater 0.5**

Supplement: Supplementary 1 — Figure. Is. Showing the PCC values for multiple treatments with values > 0.5. [file 5206758.f1.pdf]
